# Soil Microbial Resources for Improving Fertilizers Efficiency in an Integrated Plant Nutrient Management System

**DOI:** 10.3389/fmicb.2018.01606

**Published:** 2018-07-31

**Authors:** Adnane Bargaz, Karim Lyamlouli, Mohamed Chtouki, Youssef Zeroual, Driss Dhiba

**Affiliations:** AgroBioSciences, Research and Development OCP Group, Mohammed VI Polytechnic University, Ben Guerir, Morocco

**Keywords:** fertilizers, nutrient use efficiency, phosphorus, solubilization, biological N_2_ fixation, mycorrhizae, soil fertility

## Abstract

Tomorrow’s agriculture, challenged by increasing global demand for food, scarcity of arable lands, and resources alongside multiple environment pressures, needs to be managed smartly through sustainable and eco-efficient approaches. Modern agriculture has to be more productive, sustainable, and environmentally friendly. While macronutrients such as nitrogen (N), phosphorus (P), potassium (K), and sulfur (S) supplied by mineral fertilizers are vital to crop production, agriculturally beneficial microorganisms may also contribute directly (i.e., biological N_2_ fixation, P solubilization, and phytohormone production, etc.) or indirectly (i.e., antimicrobial compounds biosynthesis and elicitation of induced systemic resistance, etc.) to crop improvement and fertilizers efficiency. Microbial-based bioformulations that increase plant performance are greatly needed, and in particular bioformulations that exhibit complementary and synergistic effects with mineral fertilization. Such an integrated soil fertility management strategy has been demonstrated through several controlled and non-controlled experiments, but more efforts have to be made in order to thoroughly understand the multiple functions of beneficial microorganisms within the soil microbial community itself and in interaction with plants and mineral resources. In fact, the combined usage of microbial [i.e., beneficial microorganisms: N_2_-fixing (NF), P-solubilizing, and P mobilizing, etc.] and mineral resources is an emerging research area that aims to design and develop efficient microbial formulations which are highly compatible with mineral inputs, with positive impacts on both crops and environment. This novel approach is likely to be of a global interest, especially in most N- and P-deficient agro-ecosystems. In this review, we report on the importance of NF bacteria and P solubilizing/mobilizing microbes as well as their interactions with mineral P fertilization in improving crop productivity and fertilizers efficiency. In addition, we shed light on the interactive and synergistic effects that may occur within multi-trophic interactions involving those two microbial groups and positive consequences on plant mineral uptake, crop productivity, and resiliency to environmental constraints. Improving use of mineral nutrients is a must to securing higher yield and productivity in a sustainable manner, therefore continuously designing, developing and testing innovative integrated plant nutrient management systems based on relevant biological resources (crops and microorganisms) is highly required.

## Introduction

Global food demand is increasing rapidly and so more in developing nations where crop lands and resources hardly contribute to an efficient crop production needed to meet such an urgent demand for food. There is a need to intensify agricultural production in a sustainable manner through use of efficient agro-biosystems which consider the entire agroecosystem bio-chemical diversity and their potential to mitigate the adverse impacts of low soil fertility, abiotic stress, pathogens, and pests (Tilman et al., 2011; Timmusk et al., 2017). In this context, global food security issue will foster reliance on innovation, development, and delivery of technologies that lead to increased food production while ensuring sustainable intensification of agriculture. A number of innovative and efficient technologies has been adopted such as smart irrigation systems, smart fertilizers [i.e., controlled release fertilizer and enhanced efficiency fertilizers (EEFs), etc.], integrated fertilization, and diseases biocontrol strategies as well as diverse imaging- and sensing-based technologies that provide highly valuable information for monitoring and securing crop productivity. Agricultural microbial biotechnology through the integration of beneficial plant–microbe and microbiome interactions may represent a promising sustainable solution to improve agricultural production (Timmusk et al., 2017). For instance, advances in genomic, post-genomic, biochemistry, ecology, and symbiotic interactions of beneficial microbial strains have led to the development and commercialization of efficacious microbial products [biofertilizers, biostimulants, biopesticides, and plant growth promoting (PGP), etc.] with proven success to improve crops’ yield and adaptation to environmental changes, and inputs of carbon and energy (Lindemann et al., 2016; Umesha et al., 2018).

Today, microbial-based biofertilizers are considered to be among key agricultural components that improve crop productivity and contribute to sustainable agro-ecosystems. It is a component that aggregates a variety of microbial-based bio-products whose bioactivities are essential to stimulate and improve biological processes of the intricate plant–microbe–soil continuum (Singh et al., 2016). Different kind of soil microorganisms (especially bacteria and fungi) that exhibit PGP traits [generally identified as plant growth promoting microbes (PGPMs)] can be used for the production of efficient biofertilizers (Vessey, 2003; Lucy et al., 2004; Smith and Read, 2008; Khalid et al., 2009). Generally, microbial-based bioformulation may be classified into four types: (1) NF bacteria, (2) P solubilizing/mobilizing microorganisms, (3) composting microorganisms, and (4) biopesticides (Pathak and Kumar, 2016). Of note, in addition to their main function they are selected for, those microbial groups may all exhibit other PGP traits (i.e., phytohormones, siderophores, amino acids, and polysaccharides, etc.) plausibly contribute to an additional crop improvement.

Generally, beneficial rhizosphere microorganisms can boost plant growth via multiple regulatory biochemical pathways (categorized as direct and indirect mechanisms) that include manipulating the plant hormonal signaling, preventing pathogenic microbial strains and increasing the bioavailability of soil-borne nutrients (Van der Heijden et al., 2008; Mendes et al., 2013; Munees and Mulugeta, 2014; Verbon and Liberman, 2016; Jacoby et al., 2017). Direct mechanisms generally facilitate resource (i.e., N, P, K, and essential micronutrients) acquisition, modulate plant hormone biosynthesis, and various molecules either extra-cellularly in the vicinity of rhizosphere (i.e., siderophores) or intra-cellularly such as aminocyclopropane-1-carboxylate deaminase which facilitate plant growth and development by decreasing ethylene levels, and alleviating osmotic (salinity and drought) stress in plants (Nadeem et al., 2007; Zahir et al., 2008). Indirect mechanisms by which rhizosphere microorganisms could promote plant growth are mainly involved in decreasing the inhibitory effects of various phytopathogens through acting as biocontrol agents (Glick, 2012; Munees and Mulugeta, 2014) via antimicrobial metabolites biosynthesis (i.e., hydrogen cyanate, phenazines, pyrrolnitrin, 2,4-diacetylphloroglucinol, pyoluteorin, viscosinamide, and tensin, etc.), competition to nutrients and the elicitation of induced systemic resistance (Lugtenberg and Kamilova, 2009; Planchamp et al., 2015) which may occur due to a beneficial interaction of some rhizobacteria with plant roots resulting in plant resistance against some pathogenic microorganisms.

The positive impacts of microbial-based biofertilizers on growth and yield of staple crops may be limited to a single nutrient element such as N (i.e., due to N-fixing bacteria), but also to several nutrients [i.e., due to arbuscular mycorrhizal fungi (AMF)] (Bardi and Malusà, 2012). Moreover, the development of microorganisms’ consortium which is a polymicrobial mixture that contains several microbial strains belonging to different functional groups may strongly promote plant growth, yields, and healthy agroecosystems (Arora et al., 2011; Malusa et al., 2012). Success in constructing effective polymicrobial formulations with multiple modes of action depends on how functional, complementary, and synergic the candidate strains are (Malusa et al., 2012; Reddy and Saravanan, 2013). For example, inoculation with mixed cultures of *Penicillium* spp. and AM fungi induced positive and synergistic effects (especially enhanced plant nutrition and growth) in cereals and legumes (Kucey, 1983; Osorio and Habte, 2001; Babana and Antoun, 2007). Such positive impacts on legume crops have also been observed when co-inoculating with *Rhizobium* spp. and *Penicillium* (Downey and van Kessel, 1990; Rice et al., 2000), rhizobia with AMF (Farzaneh et al., 2009; Wang et al., 2011), *Rhizobium* and P solubilizing-bacteria (Alagawadi and Gaur, 1988), or even with the tripartite inoculation with AMF-*Rhizobium*-P-solubilizing fungus (Meng et al., 2015; Zhu R.F. et al., 2016). Multifunctional microbial consortia may also involve free-living NF bacteria as well as different PGP rhizobacteria with higher abilities to maximize plant growth, yield and efficient N uptake (Lisette et al., 2003; Wu et al., 2005; Malusa et al., 2007; Adesemoye et al., 2009; Vassilev et al., 2015).

Coincident with the scrutiny that has been given to uncovering beneficial microorganisms for optimizing their application as sustainable agriculture components, the combined use of biological, mineral, and organic resources is also increasingly gaining recognition as a promising approach. This will help elaborate efficient integrated plant nutrient management systems (IPNMSs) that address soil fertility issues, crop nutrient needs and sustainable eco-intensification. Indeed, while mineral fertilizers provide high amounts of nutrients to plants, biological resources (i.e., microbial inoculants) are key components of such IPNMS wherein both resources may synergistically co-interact to improve nutrient compositions and biological functions that plants need to grow stronger. In this regards, a limited number of studies have focused on the positive and complementary combinatory effect of using beneficial microbes for increasing the efficiency in use of mineral fertilizers (Vargas et al., 2000; Shata et al., 2007; Yasari et al., 2009; Shoghi-Kalkhoran et al., 2013; Ahmad et al., 2017). For instance, co-inoculation with *Sinorhizobium meliloti* RMP and *Pseudomonas aeruginosa* GRC2 improved growth and yield of *Brassica juncea* supplied with urea and diammonium phosphate (DAP) fertilizers (Maheshwari et al., 2010). Likewise, dual mineral fertilizer supply and inoculation with NF bacteria (i.e., *A. cholococcum*, *Azospirilum brasilense*, *Azospirilum lipoferum*, *Sinorhizobium* spp., *Burkholderia* spp., and *Pseudomonas* spp.) significantly improved yield of multiple agriculturally important crops including legumes (Gupta et al., 2002; Pandey and Maheshwari, 2007; Shata et al., 2007; Yasari et al., 2009; Shoghi-Kalkhoran et al., 2013).

Both basic and applied research on screening, designing, testing and validating potential microbial resources for their beneficial impacts on agriculture have gained global interest. Particularly, NF bacteria (both symbiotic and non-symbiotic) and P solubilizing/mobilizing microorganisms have increasingly been used as biofertilizers, and now account for more than 75% of globally marketed microbial-based biostimulants. These segments are expected to grow by 20 and 13% for the P-solubilizers and N_2_-fixers segments, respectively (Agro News, 2014; Micro Market Monitor, 2015; Novonous, 2016; Timmusk et al., 2017). Given their importance for promoting sustainable agriculture, these microbial-based biostimulants need to be more deeply explored in combination with multiple nutrient resources such as mineral fertilizers and relevant agricultural practices in order to develop effective integrated strategies that sustain crop production and soil fertility. This review aims to highlight the importance of the latter nutrients- N- and P-supplementing microorganisms in a context of promoting sustainable agriculture owing to their specific metabolic functionalities to increase use of essential nutrients (P and N) by major crops such as cereals and legumes. Furthermore, recent knowledge on the dual use of the microbial and mineral nutrient resources with peculiar emphasis on P fertilizers was presented as an example of positive IPNMS that may lead to a profitable “microbial/mineral” inputs marriage.

## Microbially-Mediated N and P in Soils

**FIGURE 1:**
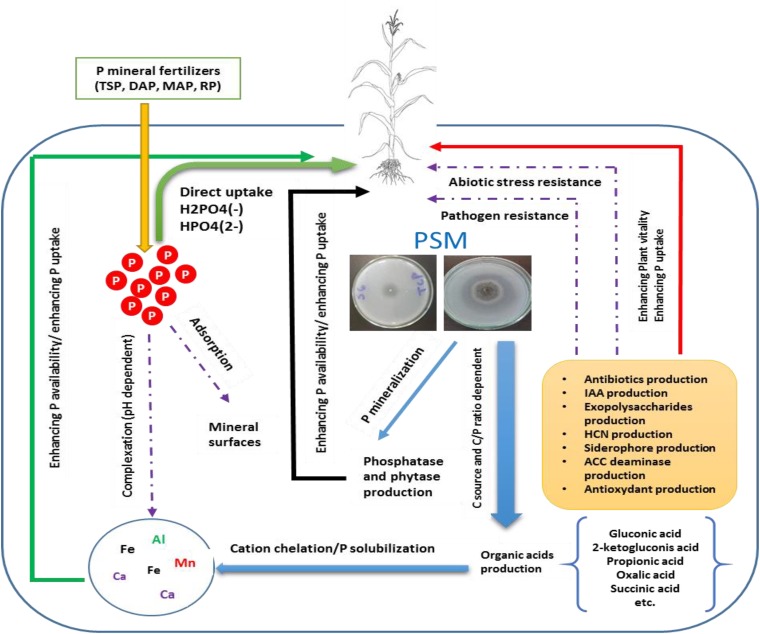
Conceptual overview illustrating the role of P solubilizing microorganisms (PSMs) in enhancing P mineral fertilizers eco-efficiency. PSM increase bioavailable P either directly by the production of low molecular weight organic acids, thus chelating through their carboxylic groups, cations attached to insoluble P, or indirectly by synthetizing bioactive molecules (phytohormones, siderophore, antibiotics, etc.) which improve plant vitality and resilience to biotic and abiotic stress and ultimately leads to better nutrients uptake and agronomic yield.

**FIGURE 2:**
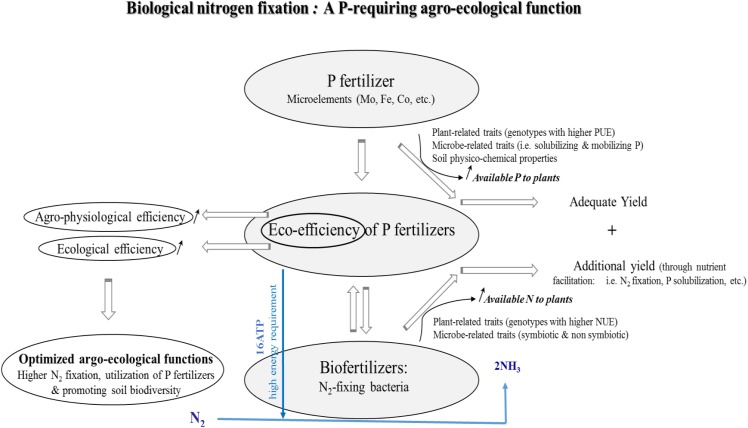
Conceptual illustration of the relationships between mineral P fertilizers and N_2_-fixing bacteria. Biological nitrogen fixation (BNF) is a process for which P is needed in relatively large amounts, especially by legumes for growth, nodulation and grain yield production. Positive interaction between P fertilizers and N_2_-fixing bacteria either symbiotic or non-symbiotic (to a lesser extent) would enhance the agronomical eco-efficiency of P fertilizers. Such a positive relationship leading to enhancing use of available P and N would also be attributed to a number of traits related to plants (i.e., above- and belowground, especially rooting) and microorganisms (i.e., P-solubilizing, phytohormones-producing, and siderophores production, etc.).

Microbial biotechnology through the exploitation of microbial resources has proved in the last 30 years to be one of the most powerful and potent tool that could provide palpable answers to address nutrient limitations (notably N and P) in most agricultural soils. Today, beneficial microbes are extensively used (as inoculants, biofertilizers, or biostimulants) to promote plant growth and to act as biological control agents. In this paper, the importance of the beneficial microorganisms belonging to NF and P-solubilizing/mobilizing groups is illustrated in **Figures 1**, **2**. The significant involvement of those two microbial groups as key drivers of N and P dynamics in soils as well as their use efficiency by plants are presumably to provide clear evidence of two inseparable microbial–rhizosphere processes as described in **Figure 2**.

### Nitrogen Fixing and P Solubilizing Microorganisms: Starting Point

Prior to the discovery of agriculturally beneficial microorganisms, especially NF bacteria, farmers used to transfer productive soils from one field to another, given it positively affected the crop productivity (Bashan, 1998). This is how bioinoculation was practiced until Boussingault in 1838 presented evidence for N fixation by legumes (e.g., clover). He showed that clover plants could gain more N than that of manure in addition to other benefits they may have on other non-legumes during crop rotation. Fifty years later, Hellriegel and Wilfarth had established the basics of the biological aspects of the legume symbiotic NF owing to the presence of nodules on the roots of *Pisum sativum* which were believed to be induced by soil bacteria (Cocking, 2009). This was better understood in 1888 when Beijerinck isolated the first *Rhizobium leguminosarum* (Evans and Russell, 1971). Then, it took less than 10 years before beneficial microorganisms are used as bioinoculants by Nobbe and Hiltner (1896). They patented what may be considered the pioneer product of all rhizobial inoculants (Patil and Solanki, 2016), launching the commercial history of microbial biofertilizers with a *Rhizobium*-based bioinoculant named “Nitrogin.” Since then, scientists from academic and private research institutions have been exploring the NF abilities of microorganisms such as *Azotobacter* and then the cyanobacteria in promoting growth of large number of plants. A few decades later, two mainly microbial based products namely “azotobakterin” and “phosphobacterin” based on *Azotobacter chroococcum* (NF) and *Bacillus megaterium* (PSB) were used in Russia and East European countries. For 1958, it was reported that about 10 million ha in Russia were treated with those microbial preparations (Brown, 1974; Rovira, 1991) and that some increase in yield was generated for various crops, though efficacy was not reproduced in other parts of the world.

With regards to P biosolubilization, evidence of the involvement of some soil microorganisms in this phenomenon is by no means novel, dating back to early 1903 (Kishore et al., 2015). It is predominantly works by Gerretsen (1948) and Pikovskaya (1984) which unfolded for the first time the ability of some microorganisms to solubilize insoluble P. This led afterward to the discovery of several P solubilizing microorganisms (PSMs) beneficial bacteria and fungi, living in close association with plants. This opened up the promise of powerful tools aimed at establishing sustainable agriculture through enhancing P use efficiency. A few years later, the microbial inoculant based on P-solubilizing *B. megaterium* (i.e., Phosphobacterin) was successfully used in Russia. Further studies however did not show the same efficiency in soils in the United States, thus concluding that there were no enough indications clearly demonstrating beneficial effects on field crops (Smith et al., 1961). After Pikovsakaya’s works, it took no less than half a century for the introduction of efficient products aimed at enhancing P use efficiency, such as “Jumpstart,” which consisted of a *Penicillium bilaii* strain isolated by Kucey (1983). As of right now, biotechnological applications relating to P biosolubilization and BNF have reached the milestone of providing efficacious products. This is come thanks to advances in both fundamental and applied sciences which provided a better understanding of the plant–microbes interactions, and a growing interest of the industrial sector.

### Biological Nitrogen Fixation, Importance, and Estimates

In general, BNF refers to a microbially mediated process by which atmospheric N_2_ is reduced into ammonia (NH_3_) in the presence of nitrogenase. Such an enzymatic conversion is found in a wide diversity of NF organisms called diazotrophs. Some diazotrophs can fix N_2_ in the free-living state, while others perform it in association with plants including endophytic (inside plant tissues) and symbiotic bacteria involving structural and physiological modifications of both microbe and plant roots in specialized structures namely nodules (Unkovich et al., 2008). For example, legumes have the advantage to associate with specific soil rhizobial bacteria (i.e., *Rhizobium*, *Bradyrhizobium*, *Mesorhizobium*, *Sinorhizobium*, and *Allorhizobium*) that can use root nodules to sequester atmospheric nitrogen as ammonia, a form of N that can then be incorporated into organic components including proteins and nucleic acids (Unkovich et al., 2008; Pankievicz et al., 2015). In symbiotic nitrogen fixation (symbiotic NF), net transfer of biologically fixed N directly from the bacteria to the host plant occurs concurrently with significant transfer of photosynthetically fixed plant carbon to the NF bacteria (Unkovich et al., 2008). Symbioses between NF bacteria and eukaryotes also include the cyanobacteria with fungi that occur in lichens, cycads and gunnera as well as actinomycetes (i.e., *Frankia*) with a variety of angiosperms like *Alnus* and *Casuarina* (Unkovich et al., 2008). However, in this review attention is paid to both symbiotic and non-symbiotic NF in an agronomical perspective.

In non-legumes such as grasses, the ability to fix N_2_ has been extensively studied and remarkable advances have been achieved from the cell to the farm context. Several NF bacteria which also exhibit PGP properties have been identified as non-symbiotic NF bacteria of grass species, especially cereals in which they significantly increase plant vegetative growth and grain yield as described further in the paper (Malik et al., 2002; Kennedy et al., 2004). Among them, *Beijerinckia*, *Azotobacter, Azospirillum, Herbaspirillum*, *Gluconacetobacter*, *Burkholderia*. *Clostridium*, *Methanosarcina*, and *Paenibacillus* are well-known. Unlike rhizobia that lead to the formation of root nodules with their legume hosts, non-symbiotic NF bacteria are either rhizosphere free-living or endophytic (inside living tissues) with the ability to proliferate owing to energy and nutrients derived from plant roots (Unkovich et al., 2008; Pankievicz et al., 2015). It is worth mentioning that, unlike symbiotic NF, non-symbiotic NF (which is commonly referred to as associative N_2_ fixation) does not involve a direct controlled exchange of N and C between bacteria and plant hosts.

Accurate determination of global inputs of biologically fixed N has always been a challenge (Peoples and Herridge, 2000; Herridge et al., 2008). Because data on area and productivity of NF legumes and non-legumes are almost impossible to obtain, BNF is difficult to measure. Methodological constraints used to estimate N_2_ fixation are also a major component of this challenge (Roger and Ladha, 1992; Herridge et al., 2008; Unkovich and Baldock, 2010; Ladha et al., 2016). In legume crops, published estimates by Peoples et al. (1995) reported BNF to be in the range of 100–300 kg N ha^-1^. Other studies (i.e., Herridge et al., 2008) pointed out the rhizobia–legume symbiotic associations to be the most important NF biosystems, contributing with average N_2_ fixation estimates of 227 kg N ha^-1^ annually. As per crop biomass, Peoples et al. (2009) estimated the legume rhizobial symbiosis to fix the equivalent of 30–40 kg N per ton of shoot dry matter and that the most efficient NF crops like soybeans can accumulate up to 200 kg N ha^-1^ annually. Similarly, faba bean, which is a commonly grown grain legume used as a valuable protein source and energy for food and feed, has high reliance on N_2_ fixation estimated at up to 100–200 kg N ha^-1^ (Jensen et al., 2010). On the other hand, large-scale data on non-symbiotic NF estimates are scarce except for staple cereal crops such as maize, rice, and wheat. Recently, a 50 year assessment study reported non-symbiotic NF estimates for maize, rice, and wheat production systems to contribute to an average of 15.5 kg ha^-1^ (Ladha et al., 2016). On a per hectare basis, estimates indicated that non-symbiotic NF contributed 13, 22, and 13 kg to the N harvest of maize, rice, and wheat, respectively, in which the efficiency of N contribution to the crop was assumed to be 80% (Ladha et al., 2016). In rice cropping system, Ladha et al. (2016) found comparable fixation rates with the value reported by Bouwman et al. (2013), however much lower than those estimated by both Ladha et al. (2000) and Bei et al. (2013). Likewise, fixation rates up to about 20 kg N ha^-1^ were estimated through a long-term study with wheat; rates that were at least twofold higher than estimates (5–10 kg N ha^-1^) from some studies (Herridge et al., 2008).

### Microbial Solubilization and Mobilization of Phosphorus

Phosphorus is a fundamental mineral nutrient for agricultural and crop development and essential in vital physiological processes (Krishnaraj and Dahale, 2014). As a matter of fact, that agricultural production has almost tripled since 1950 is widely attributed to the introduction of mineral fertilizers including P fertilizers into agroecosystems (Ekardt, 2016). P is known to promote root development, rapid plant maturity, seed production, improve BNF, water use efficiency, and resistance to diseases (Murrell and Munson, 1999). It is also fundamental in vital physiological processes including, energy transfer and storage, photosynthesis, cell division and seed formation which are all energy dependent biological process controlled by two intracellular P-containing molecules; ADP (adenosine diphosphate) and ATP (adenosine triphosphate; Krishnaraj and Dahale, 2014).

As per plant and soil content, P represents ∼0.2% of total plant dry weight and 0.05% (w/w) of soil content of which only a small fraction is bioavailable for plants (Alori et al., 2017). In soil solution, the bioavailable form of P rarely exceeds 10 μM and plants must possess specialized transporters and complex efflux system providing efficient distribution of this nutrient between all plant compartments (Schachtman et al., 1998). Such a lower P concentration in soil solution makes this nutrient very limiting for plant growth giving that crop production requirement for P is relatively sizable (Bhat et al., 2017). Further, P availability is a pH-dependent process and fixation and precipitation phenomena may occur and thus reduce P availability and consequently efficiency of P fertilizers. For example, in calcareous soils, P fertilizer use efficiency is severely hampered due to precipitation and adsorption (Sanders et al., 2012). Therefore, one of the key challenges faced by today’s agriculture entails developing solutions for optimizing or enhancing the bioavailability of P knowing that productivity needs to increase by several fold to meet increasing demand of growing population. Soil microorganisms exhibiting beneficial traits responsible for bio-solubilization of insoluble forms of P are presumably needed in most agricultural soils where P use efficiency by plants has to be enhanced. In this context, a large number of greenhouse- and field-based studies (i.e., Rodrıìguez and Fraga, 1999; Khan et al., 2010; Wu et al., 2012; Sawers et al., 2017) have demonstrated that application of PSM and AMF is associated with higher plant P uptake and increased yield of many vegetable and cereal crops. In addition to underlining the role of these microorganisms in enhancing P bioavailability, this review provides evidence to showcase the advantage that could be gained from the combinatory use of both P mineral fertilizers, PSM and AMF.

### Phosphate Solubilizing Microorganisms (PSM)

A number of genera among bacteria (i.e., *Pseudomonas*, *Bacillus*, *Azotobacter*, and *Bradyrhizobium*), fungi (i.e., *Penicillium* and *Aspergillus*), actinomycetes (i.e., *Streptomyces*), and algae are all capable of solubilizing P-metal complex to release bioavailable P in the form of orthophosphate through specific mechanisms implying mainly organic acids, siderophore production, and phosphatase enzymes playing a key role in hydrolyzing organic P forms. Overall, PSM could contribute in promoting plant growth through enhancing P use efficiency directly through exudation of organic acids and P-hydrolyzing phosphatase enzymes and thus enhancing the bioavailable P pool, or indirectly through the production of phytohormones, antifungal compounds, toxin-resistance compounds, and other high value bioactive molecules which could help building robust shoot/rooting system, specially under biotic and abiotic constraints (**Figure 1**).

Despite the variety of mechanisms involved in P solubilization, organic acids are the main contributors (Khan et al., 2007; Chen et al., 2015; Wei et al., 2018). Secretion, quantitatively and qualitatively, of organic acids by beneficial microorganisms is mainly gene-dependent but could also be influenced by the ecosystem environmental properties (Zhen et al., 2016). For example, N and C soil content may have a direct impact on the nature of the organic acids produced, the nature of C source could affect the bio-solubilization process, and high C/P ratio seems to increase the production of organic acids while both C/N and N/P may affect microorganism’s development (Zhen et al., 2016). It is also important to note that P solubilization efficiency depends more on quality rather than quantity of organic acids and P sources (Scervino et al., 2010). The latter authors, when comparing P solubilizing properties of tow fungal strains (*Talaromyces flavus* and *Penicillium purpurogenum*), found that both strains exhibit equal solubilization potential, although *T. flavus* produced four times less organic acids than *P. purpurogenum*. This finding, which is in line with a study by Zhen et al. (2016), indicated a clear relationship between organic acids profile and the source of P regarding the bio-solubilization process.

It is worth mentioning that PSM can produce a number of organic acids (i.e., acetic acid, gluconic acid, glucuronic acid, butyric acid, fumaric acid, citric acid, lactic acid, propionic acid, succinic acid, oxalic acid, and valeric acid) with 2-ketogluconic acid and gluconic acid are the most common ones in gram negative bacteria (Krishnaraj and Dahale, 2014). Organic acid implication in P solubilization is often attributed to lowering the pH and cations chelating properties (Zeroual et al., 2012; Behera et al., 2017). The acidification of microbial cells perimeter leads to the release of P anion by substitution of H^+^ and Ca^2+^ (Trivedi and Sa, 2008; Behera et al., 2017). Nevertheless, other studies reported no correlation between P solubilization and pH value implying that other mechanisms might be involved in this phenomenon. This includes mechanisms such as the release of protons after ammonium assimilation by microbial cells, the production of inorganic acids (i.e., sulfuric and nitric acids) and the production of specific enzymes acting on amphiphilic fatty substances (Alori et al., 2017).

In addition to microbial solubilization of mineral P, organic P mineralization through the action of microorganisms plays also a vital role in P cycling, giving that organic P content in soil (mostly in the form of inositol polyphosphates) can represent between 30 and 50% of the total P (Shen et al., 2011). The mineralization process is widely governed by specialized P-hydrolyzing enzymes produced by microbes such as phytases and phosphatases which are a non-specific exo-enzymes principally produced by fungi and bacteria (Sato et al., 2015). P mineralization is largely attributed to acid phosphatases which dephosphorylate phosphor-ester compounds and phosphoanydrid bonds of organic compounds (Alori et al., 2017). In addition to their positive contribution in enhancing P bioavailability, soil microorganisms mediating P availability possess other substantial attributes of agronomic interests including production of phytohormones, enhancing the resilience to biotic and abiotic stress through the production of specific compounds (antifungal compounds), and the regulation of key metabolic pathways (Khan et al., 2007; Popavath et al., 2008; Sharma et al., 2013).

### Arbuscular Mycorrhizal Fungi (AMF)

Additionally to PSM, AMF are also key microbial component of agro-systems giving they are the most widespread symbiosis on earth (concerns more than 80% of terrestrial vascular plants) which is defined by a symbiotic relationship involving a bidirectional exchange of nutrients between the two organisms (Wang et al., 2017). Their contribution to P mobilization and uptake could be substantial and may represent in some case (depending on soil nature and P treatment) up to 80% of total P uptake (Li et al., 2006). A study by Schnepf and Roose (2006), based on a mathematical model to quantitatively evaluate the contribution of AMF hyphae to P acquisition by plants, demonstrated that plants may depend exclusively on mycorrhizal pathway for P nutrition. Morphologically, AMF act through their mycelium network as an extension of the rooting system enabling the plants to scavenge nutrients far beyond the rhizosphere boundaries and this mechanism presumably prevails in the specific case of P deficiency (Wang et al., 2017). In this regard, AMF provide an additional P uptake pathway (AMF pathway) with arbuscules being in most cases the symbiosis interface, thus sidestepping the direct uptake by root epidermis which is often quickly obstructed with the formation of a downsized depletion zone resulting from brisk absorption of P from soil solution (Shen et al., 2011). AMF hyphae have a high affinity for inorganic P and due to their reduced diameter compared to roots they can explore inaccessible soil pores and enhance translocation of inorganic P (Bago, 2000). Indeed, P is absorbed in the form of polyphosphates by AM hyphae, then transported to intra-radical hyphae through the vacuole where it is cleaved (Konvalinková et al., 2017). Additionally, it seems that through the evolution, plants have acquired specific P transporters that have been identified for various species, including rice, tomato, potato, barely, and clover (Paszkowski et al., 2002; Konvalinková et al., 2017; Wang et al., 2017).

However, until now, and even if the ability of fungal hyphae to exudate acid phosphatase enzymes and organic acids has been confirmed (Sato et al., 2015), the magnitude in which AMF can directly contribute in enhancing P availability in comparison to PSM and roots is still not well-defined. That being said the general consensus is that AMF are involved in P solubilization through a synergic relationship with PGPM. Indeed, recent studies (Taktek et al., 2017) have shown that the PGP bacteria associated to the AMF hyphae known as hyphobacteria could play a vital role in mycorrhizal symbiosis which strongly suggest that there is an AMF/bacteria specificity. In fact, rhizobacteria could benefit from fungal exudates as a source of nutrients while hyphobacteria provide growth factors that stimulate both mycorrhizal symbiosis and plant development (Taktek et al., 2015). Additionally, hyphobacteria can abundantly generate biofilm-like structures which enhance nutrients biodynamic (Iffis et al., 2014). Furthermore, Cruz and Ishii (2012) hypothesized that the entophytes closely associated with AMF could be involved in nutrient bioavailability. Indeed, those authors successfully isolated three endobacetria (*Bacillus* sp., *Bacillus thuringiensis*, and *Paenibacillus rhizospherae*) from *Gigaspora margarita* spores that exhibited multiple PGP properties including P solubilization, ethylene production, nitrogenase activity, and antagonism toward soil-borne pathogens. Dual positive effects of AMF and their associative endobacteria with regards to facilitation of P uptake under P-limiting conditions were evidenced by Battini et al. (2017). This study highlighted the contribution of 10 bacteria isolated from AMF spores in enhancing P availability and improved hyphae elongation which were both attributed to phytohormones production.

Moreover, it was reported that some AMF spores belonging to the *Gigasporaceae* are habitats of several endophtytic bacteria. For example, the endophtytic bacteria *Glomeribacter gigasporarum* which was previously assigned to the *Burkholderia* genus according to 16S rRNA sequencing, lives inside the mycorrhizal spores and cannot complete their life cycle otherwise (Artursson et al., 2006). This is clear evidence demonstrating that what we thought as a bipartite symbiosis is in some specific cases a complex tripartite symbiosis. According to Ghignone et al. (2012), *G. gigasporarum* have very limited sugar metabolism and depends exclusively on its AMF host for carbon and P supply, whereas the endophytic bacteria produces vitamin, antibiotics, and specific molecules with toxin resistance properties that enhance the host resistance toward indigenous microbes. The specific role of AMF-associated bacteria is, however, still not well-known and future research should focus more on microbial dynamic around and within the mycorrhizal symbiosis components. In addition, AMF have other beneficial impacts on the whole plant/soil system (Berruti et al., 2016; e.g., resilience to abiotic and biotic stress, improvement of soil texture, enhancing microbial activity such as BNF as detailed further in the paper), which make them key components of productive and substantial agroecosystems that should be taking into consideration when implementing nutrients management programs.

### Phosphorus Availability Enhances Biological Nitrogen Fixation

Both symbiotic and non-symbiotic BNF constitute a major input of N in agroecosystems and may provide an ecologically acceptable complement or substitute for mineral N fertilizers (Peoples et al., 1995, 2009; Herridge et al., 2008; Lazali and Bargaz, 2017). However, BNF is often limited under low soil nutrient availability, notably P that is required at sufficient rates during the BNF process (Schulze and Drevon, 2005; Alkama et al., 2012). More particularly, high P requirements were found to be more critical in N_2_-fixing (NF) legumes than in non-symbiotic plants whose growth rely on mineral N sources (Serraj and Adu-Gyamfi, 2004; Schulze and Drevon, 2005; Sulieman and Tran, 2015). Generally, in soils containing only a small fraction of P that is readily available, plant growth and associated-metabolic pathways would be greatly impaired, which makes necessary the application of mineral P fertilizers to replenish the soil as to immediately satisfy plant requirements for better growth and yield (Richardson et al., 2009).

Much information is available about the important role of mineral P fertilization in plant growth processes, including nutrient uptake (Ca, Mg, Zn, Cu, Mn, and Fe, etc.), photosynthesis, root development, root hair formation, nodulation, BNF, and particularly as an energy source for the latter biological process (Israel, 1987; Drevon and Hartwig, 1997; Gordon et al., 1997; Nielsen et al., 2001; Hogh-Jensen et al., 2002; Nziguheba et al., 2016). Plant N nutrition, with emphasis on legume crops (i.e., lentils, faba bean, common bean, and cowpea, etc.) may be affected positively in response to an adequate P nutrition that has positive consequences on robust root systems, vigorous seedlings, ammonium assimilation into amino acids, and ureides, as well as synthesis of mitochondrial and symbiosome membranes for functional NF nodules (Fageria et al., 1995; Schachtman et al., 1998; Nyoki and Ndakidemi, 2014a; Tairo and Ndakidemi, 2014; Sulieman and Tran, 2015). This is of particular importance for NF legumes whose nodule formation, nodule functioning, and the energy costs related to NF greatly depend on the P status in plant and nodule tissues (Vardien et al., 2016). Moreover, the fact that root-nodules are strong P sinks, with nodule P concentrations often exceeding those of roots and shoots also indicates the important role of P in the legume symbiosis processes (Schulze and Drevon, 2005; Bargaz et al., 2012; Nyoki and Ndakidemi, 2014a).

Other traits related to extensive rooting system and their spatial distribution, hyper-nodulation, root exudates, rhizosphere acidification, and heterogeneity are among the most important plant-related belowground traits that contribute to higher nutrient use efficiency (**Figure 2**). Regarding P, these traits may substantially contribute in alleviating the sensitivity of NF plants to low P availability through ensuring large amount of P-dependent carbon and energy turnover required during the NF process (Schulze, 2004; Serraj and Adu-Gyamfi, 2004; Schulze and Drevon, 2005). Moreover, exploiting beneficial microbial traits involved in higher P solubilization would positively influence P uptake in addition to multiple advantages attributed to the production of plant growth-promoting substances which could indirectly influence the efficiency of BNF (Kucey et al., 1989; Afzal et al., 2010). For example, dual inoculation of soybean plants with both a P-solubilizing (*Bacillus*) and NF (*Bradyrhizobium*) strains improved symbiotic traits related to growth of nodules and roots, aboveground biomass, total N and grain yield (Ming et al., 2003; Afzal et al., 2010). However, despite positive responses on improved growth, nutrient use efficiency (N and P), and stable yield, all were demonstrated due to microbial application and mineral supply, co-application of multipurpose microbial strains, host plant species, and nutrients sources may generates a highly intricate plant–soil–microbe interactions that need to be profoundly deciphered in order to optimize the agronomical functions they were designed for.

## Synergistic Use of Mineral P and N_2_ – Fixing Bacteria to Yield Better and Sustainably

As mentioned above, the reliance of legume-based cropping systems on biological NF is challenged by the fact that many legumes are sensitive to a broad spectrum of environmental constraints (notably P with 40% of the land is limited by low P availability) and this leads to great variation in terms of growth, nodulation, and thus N_2_ fixation rates in legume crops (Vance, 2001; Carlsson and Huss-Danell, 2003; Deng et al., 2005; Hauggaard-Nielsen et al., 2010). Under stressful conditions, legume and non-legume NF crops may lose the distinct advantage of an unlimited source of biological N (Vance, 2001; Shenoy and Kalagudi, 2005), and that the potential of such highly valuable cropping systems to efficiently use nutrients needs to be preserved.

### In Legume Crops

Enhanced P and N uptake by legume crops using beneficial NF and P-solubilizing microorganisms has been adopted worldwide. However, such a biological approach needs to be further optimized for better use of mineral resources such as P, better crop productivity and resiliency to abiotic and biotic constraints. In this context, and given that BNF greatly relies on P availability in soils, efficient root uptake and use of P would stimulate the functioning of the legume symbiosis in terms of N nutrition such as in the lentil-*R. leguminosarum* association whose N requirement might be secured up to 80% through symbiotic NF according to The Saskatchewan Pulses Growers (Pulses Crop Development Board). Coincident with the efficient use of adequate amounts of P fertilizers required for optimal plant productivity, aboveground N requirement may then be promoted by using effective NF strains and relevant crop–microbe biosystems that are efficient in contributing to sustainable intensification of agriculture. Although not fully exploited, some inoculants can substantially improve plant uptake of essential nutrients and thereby increase use efficiency of applied mineral and organic fertilizers (Adesemoye and Kloepper, 2009). In this context, microbial inoculants such as rhizobia have been widely used to promote BNF in intensive farming systems that require adequate amounts of fertilizers (i.e., P and K) to realize high yield and socioeconomic benefits to farmers (Sanchez et al., 1997; Gruhn et al., 2000).

Several reports revealed that rhizobial inoculation of many legume crops (such as cowpea, chickpea, soybean, common bean, etc.) supplemented with P fertilizer improved the uptake of N, P, K, Mg, Ca, and Na (Messele and Pant, 2012; Makoi et al., 2013; Nyoki and Ndakidemi, 2014b). Likewise, Verma and Singh (2008) previously reported improved plant symbiotic performance (biomass, number, and nitrogenase activity of nodules) and yield in mung bean inoculated with *Rhizobium* and supplied with P (45 kg P_2_O_5_ ha^-1^). However, and despites P has been demonstrated to be essential for the rhizobial symbiosis establishment and functioning, there are so far fewer reports on the effects of combined use of P fertilizers and rhizobial inoculation that could produce additional benefits to the symbiosis performance and host feedback responses. Recently, a study by Kyei-Boahen et al. (2017) found that dual application of rhizobia inoculants together with mineral P fertilizer improved cowpea-*Bradyrhizobium* symbiosis agronomic efficiency compared to either inoculant or P applied alone. In addition to a positive influence that P may have on the rhizobia efficiency (higher N content in shoots and seeds) and yield component (grain yield and plant biomass), application of this nutrient was also been demonstrated to boost the effectiveness and efficiency of the indigenous rhizobia population (Kyei-Boahen et al., 2017). Those measured improvements in growth and productivity were achieved under medium P fertilization level (up to 40 kg P ha^-1^), which is in line with previous studies that succeeded to physiologically explain advantageous mutualistic benefits in response to P supply and rhizobia inoculants (Bambara and Ndakidemi, 2010; Makoi et al., 2013; Tairo and Ndakidemi, 2013; Nyoki and Ndakidemi, 2014a). Moreover, the impacts of a slow release plant fertilizer (containing NPK “19–6–12”) on symbiotic and plant phenotypic traits were investigated through multiple controlled-condition experiments (Simonsen et al., 2015). These authors found that rhizobial isolates (*Ensifer meliloti* and *Ensifer medicae*) from a fertilized-field soil conferred higher mutualistic benefits with *Medicago lupulina*. The combined-micronutrients plant fertilizer was generally beneficial for rhizobia growth compared to isolates from unfertilized field soil. Simonsen et al.’s (2015) finding concords with several previous studies on rhizobia growth and effectiveness that promoted legume symbiosis functioning owing to increased availability of multiple mineral nutrients, notably P (Gates and Wilson, 1974; Asimi et al., 1980; Israel, 1987).

With regards to P application and BNF, Reed et al. (2011) through a long term agroforestry-based study demonstrated that the efficiency of N-fixing community was found to be tightly linked to P supply which indicates a tight coupling of N and P demands. Moreover, based on an intercropping cereal–legume study, Tang et al. (2016) also concluded that P fertilization is presumably driving soil microbial communities since it resulted in a higher abundance of bacterial and fungal communities. Conversely, long-term N addition was reported to suppress the mutualistic benefits of the legume–rhizobia associations (Carroll and Gresshoff, 1983; Imsande, 1986; Streeter and Wong, 1988; Weese et al., 2015). This response, according to Kiers et al. (2007) and Coelho et al. (2009), could directly be attributed to decreased rhizobia abundance in soils and reduced selective pressure from legumes to maintain beneficial partners. Generally, it was reported that long-term N rather than P fertilization may decrease significantly the abundance of functional bacterial groups, such as NF bacteria, ammonia oxidizing bacteria, and AMF (Avio et al., 2013; Berthrong et al., 2014). For example, a field-based study on 8-year-old alfalfa monocultures demonstrated that long-term P fertilization influenced soil fungal and bacterial diversity rather than the P-mobilizing AMF community (Beauregard et al., 2010). Coherently, Zheng et al. (2017) demonstrated that a long-term inorganic P fertilization had no effect on P-solubilizing bacterial communities, in contrast to a long-term N fertilization that decreased their abundance. This decrease was attributed mainly to soil acidification, total N and P release thus explaining a lower demand for functional Pi-solubilizing bacteria populations.

Moreover, combinatory use of the rhizobial symbiosis and P fertilization has been shown to help ensure yield stability under stressful conditions such as salinity owing, among other factors, to stimulating plant–defense mechanisms coupled with adequate nodulation, plant biomass, protein content, grain yield, and other growth variables (Ankomah et al., 1995; Onduru et al., 2008; Dekhane et al., 2011; Musa et al., 2011; Nyoki and Ndakidemi, 2013; Bargaz et al., 2016; Kyei-Boahen et al., 2017). In this context, studies by Abd El-Hamed et al. (2012) and Khan et al. (2013) highlighted that mineral P fertilization may mitigate salinity stress effects, and that was demonstrated by the improved wheat growth concurrently with increasing N, P, K, and Zn uptake. Improved salt tolerance was also reported in common bean (Bargaz et al., 2016) and chickpea (Sadji-Ait Kaci et al., 2017) under P fertilization whose adequate application could be considered a promising strategy to alleviate deleterious salinity effects and to stabilize productivity of such both NF and protein-rich grain legumes. Other studies also reported pronounced salt stress alleviation in plants in response to a combined application of P with K, indole acetic acid (Kaya et al., 2013), organic P (Abd El-Hamed et al., 2012), and humic acid (Çimrin et al., 2010). In those studies, application of P fertilizer was coupled with improved plant growth performances owing to a number of physiological changes (stimulation of proline, glycine, soluble sugars, and antioxidants, etc.) that contributed to osmotic adjustment under salinity stress conditions (Bekheta et al., 2009; Shahriaripour et al., 2011; Abdelhamid et al., 2013; An and Liang, 2013). In response to other abiotic constraints such as elevated carbon dioxide (as a consequence of climate change), Sumit et al. (2017) recently found that a combined application of P and a NF bacterium (cyanobacterial inoculant) enhanced rooting and symbiotic traits related to nodulation, N_2_ fixation, and uptake in cowpea crop.

### In Non-legume Crops

Unlike legumes, members of the *Poaceae* family do not naturally form symbiotic NF associations, but they can derive a substantial part of their N through non-symbiotic associations with free, associative and endophytic NF bacteria. In non-leguminous crops like cereals, the optimization of NF ability has long been a major goal of plant scientists not only to make cereals self-sufficient in N nutrition (Galal et al., 2000; Viviene and Dakora, 2004), but also to achieve better efficiency in use of major soil and fertilizer nutrients such as P. Several studies have demonstrated improved yield of numerous cereal staple crops in response to a mineral fertilization (NPK) and inoculation with a number of non-symbiotic NF bacteria that exhibit multiple PGPR traits (*A. chroococcum*, *A. brasilense*, *A. lipoferum* and some species of *Burkholderia*, *Pseudomonas*, *Sinorhizobium*; (Gupta et al., 2002; Pandey and Maheshwari, 2007; Shata et al., 2007; Yasari et al., 2009; Shoghi-Kalkhoran et al., 2013). Inoculation with non-symbiotic NF bacteria that simultaneously function as PGPR is likely a worldwide dream of developing sustainable nutrient sources (Adesemoye and Kloepper, 2009), notably N and P.

Dual P-based mineral fertilization and inoculation with non-symbiotic NF bacteria still yet not well-documented, and particularly under multiple abiotic- and biotic-related factors that have to be controlled. As for bacterial component and besides it may have higher abilities to efficiently use atmosphere N, stimulate P availability, root P uptake, produce growth stimulating phytohormones, bacteria resiliency to environmental conditions, adaptation to mineral fertilizer physico-chemical properties are presumably highly needed key microbial traits that are required to secure synergistic interactions with plant hosts. Beneficial rhizobacteria may involve specific mechanisms to tolerate stressful conditions such as saline soils in which *Azotobacter* species, for example, may proliferate up to 10^6^ cells per gram soil (Whipps, 2001) and are able to mitigate high temperature and acidity levels (Chennappa et al., 2016). Large number of bacteria belonging to numerous rhizobacteria genera including *Pseudomonas*, *Flavobacterium*, *Bacillus*, *Arthrobacter*, *Rhizobium*, *Azospirillum*, *Halomonas*, *Chromohalobacter*, *Salinivibrio* were characterized for their abiotic stress tolerance (Tripathi et al., 2002; Ahmad et al., 2005). However, little information is available on evaluating NF bacteria with adaptive traits to chemical fertilizer and whether they may potentially be used for implementing novel integrated plant nutrition approach and commercial benefits.

Maheshwari et al. (2010) used two adaptive bacterial strains (*Sinorhizobium* RMP1 and *Pseudomonas* GRC2) whose impacts on growth and yield of *B. juncea* were positive when co-applied with DAP and urea fertilizers. Co-inoculation with the latter NF and P-solubilizing strains were found to be effective on *Brassica* crop in terms of growth performances under half dose of N and P fertilizers (Maheshwari et al., 2010). Those findings are in line with earlier results by Pandey and Maheshwari (2007) that co-inoculation with *Sinorhizobium* and *Burkholderia* sp. enhanced growth of pigeon pea. This is in accordance with a study by Mohiuddin et al. (2000) on the profitability of integrated use of microbial-based biofertilizers and NPK fertilizers to obtain high grain yields in wheat. Another study based on pot experiments demonstrated that bacterially impregnating DAP and urea granules with the PGPB *Bacillus* sp. strain (KAP6) (slurry of strain and compost) enhanced growth, yield, photosynthetic rate, and nutrient use efficiency of wheat supplied with N-containing fertilizers such as urea and DAP (Ahmad et al., 2017). Nevertheless, at a large scale, there is no evidence of such interesting strategy of impregnating mineral fertilizers with plant growth-promoting bacteria, and particularly for NF bacteria and PSM that have been attracting worldwide attention.

Giving that most cereal-grown soils are N-deficient and that production of cereal crops such as wheat, barely, maize and rice greatly relies on N and P applications, integrated use of non-symbiotic NF bacteria and rational mineral nutrition would positively impact cereal yield components. As for microbially mediated N, it is now evident that using effective non-symbiotic NF soil bacteria (i.e., *Azotobacter*, *Azospirillum*, and *Glucanacetobacter*, etc.) that have the potency to supplement significant amounts of N would be a wiser alternative (Bashan and de-Bashan, 2010; Wani et al., 2013; Sahoo et al., 2014). Meanwhile, plant genotype is presumably a key factor that also control benefits derived from the non-symbiotic NF such as in wheat, oat and maize according to Lana et al. (2014). Other field experiments attributed the observed increase in productivity of rice inoculated with *Azotobacter* and *Azospirillum* spp. and supplied with recommended or reduced N to be genotype-driven (Kanungo et al., 1997; Choudhury and Kennedy, 2004; Singh, 2006; Pedraza et al., 2009; Zaki et al., 2009). In addition, carbon allocation (quantitatively and qualitatively) to roots and rhizodeposition are presumably important plant-related factors that may plausibly control above- and below-ground plant biomass. Those factors may modify rhizosphere traits and shape the rhizosphere microbial community (Hernández et al., 2015) that may include non-symbiotic NF bacteria, particularly via the development of robust rooting system and associated rhizosphere-induced changes including plant–microbe mutualism efficiency, BNF (Schulze and Drevon, 2005; Latati et al., 2014, 2016; Bargaz et al., 2017), soil respiration (Ibrahim et al., 2013; Latati et al., 2014), rhizosphere acidification, and P availability (Betencourt et al., 2012; Latati et al., 2016).

**FIGURE 3:**
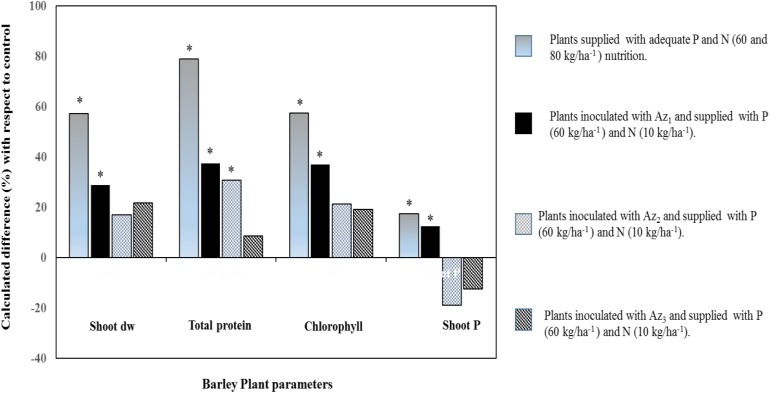
Percentage difference of four barley plant agro-physiological parameters (shoot dry weight, protein content, chlorophyll index, and shoot P) with respect to control. *Gray bars*: barley plants supplied with recommended P and N (60 and 80 kg/ha) rates. Bars that are *Black, gray, and blackhatched*: barley plants inoculated with three different non-symbiotic NF strains (Az_1_, Az_2_, and Az_3_) supplied with P (60 kg/ha) and deficient in N (10 kg/ha). Asterisks denote significant difference (*p* < 0.05) with regard to control.

All these rhizosphere-induced changes could tightly be linked to leaf photosynthetic activity such as in wheat, pea, maize, and tomato wherein an estimate of up to 60% of the photosynthesis-fixed C is belowground-translocated and that root-associated microorganisms can metabolize or use it for the benefit of plant growth and the rhizosphere microbiome (Morgan et al., 2005; Hernández et al., 2015). Coherently, stimulated photosynthesis activity was observed in barely plants inoculated with a free NF bacterium (*Pseudomonas* sp.), supplied with sufficient amount of P [triple superphosphate (TSP)] fertilizer and deficient amount of N (**Figure 3**). An increased plant biomass and chlorophyll content (especially with the strain Az_1_, **Figure 3**) may be attributed to an effective absorption of nutrients (mainly N as attested by higher protein content), but also by the capacity of this strain to synthesize indole acetic acid that could positively impact secondary roots proliferation (Spaepen et al., 2008; Baset Mia et al., 2010). Coherently, findings by Zhang et al. (1997), Han and Lee (2005), Shoghi-Kalkhoran et al. (2013), and Boisvert (2014) reported stimulation of photosynthetic activity in response to inoculation with non-symbiotic NF rhizobacteria that provide biologically fixed N and beneficial growth promoting substances like indole acetic acid, gibberellin acid, kinetin, riboflavin, and thiamine.

**FIGURE 4:**
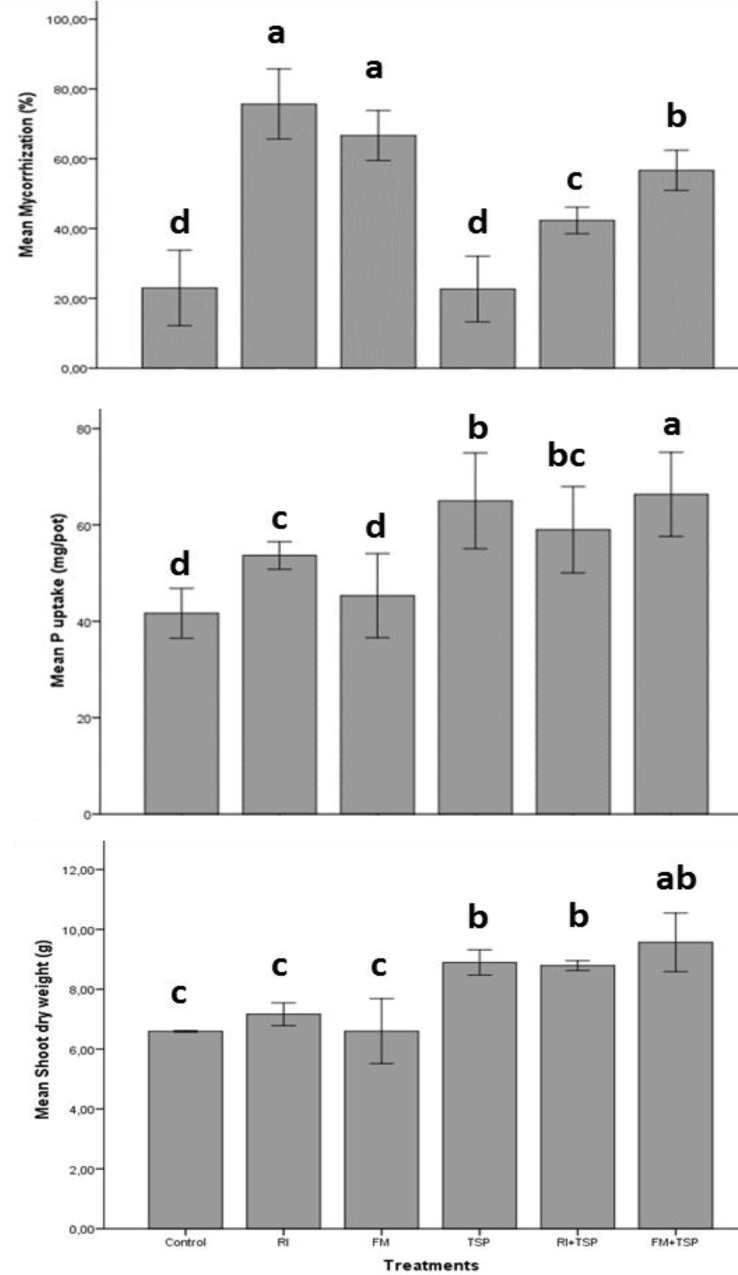
Effect of inoculation with a Moroccan native AMF species [*Rhizophagus intraradices* (RI) and *Funneliformis mosseae* (FM)] on root mycorrhiza colonization percentage, shoot dry weight (g) and P uptake (mg/pot) of maize with and without fertilization with triple superphosphate (TSP) (60 kg P/ha). Columns denoted by a different letter, differ significantly at *p* < 0.05.

Overall, it is evidenced that non-symbiotic NF bacteria improve growth, and especially functional root traits with positive consequences on uptake of water and mineral nutrients as exemplified with *A. brasilense* that promote uptake of the three essential nutrients (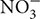, K^+^, and 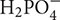) into major crops like corn, sorghum, and wheat (Okon and Kapulnik, 1986; Murty and Ladha, 1988; Okon and Hzigsohn, 1995; Saubidet et al., 2000; Riggs et al., 2001; Viviene and Dakora, 2004). In a maize inoculated with *Herbaspirillum seropedicae* and supplied with mineral fertilization (NPK) enhanced use of nutrients [especially P (30%) compared to N (11%) and K (17%)], biomass and leaf parameters compared to plants supplied with NPK fertilizer alone (Baldotto et al., 2012). Similar results were reported in **Figure 3** that showed an increased shoot P accumulation (indicating higher P fertilizer use efficiency) in barely plants inoculated with a NF and P-solubilizing Pseudomonas bacterium (strain Az_1_) compared to other tested strains. This important P uptake would have relied upon plant-induced changes, especially root growth whose nutrient absorptive capacity could be augmented owing to associated non-symbiotic NF bacteria (such as *Pseudomonas*, *Azospirillum*, *Azotobacter*, *Sinorhizobium*, *Bacillus*, and *Glucanobacter*, etc.) with multifunctional abilities other than only improving both N and P nutrition (Panwar and Singh, 2000; Kumar and Singh, 2001; López-Ortega et al., 2013; Delaporte-Quintana et al., 2017). Furthermore, the observed root P uptake improvement (Az_1_, **Figure 3**) correlated with a specific plant P translocation pattern (higher intracellular P in shoots as compared to that in roots) and the higher ability of the latter strain to solubilize tri-calcium P. Organic acids, among other factors, may enable roots to access readily available P owing to intense acidification at the root vicinity such as in wheat and tomato seedlings supplied with insoluble calcium phosphate and inoculated with a NF bacterium (*Gluconacetobacter diazotrophicus*; Sevilla and Kennedy, 2000; Crespo et al., 2011; Delaporte-Quintana et al., 2017). Moreover, Species belonging to several bacterial genera such as *Azosporillum*, *Azotobacter*, *Rhizobium*, and *Klebsiella* were reported to exhibit a higher ability to solubilize tri-calcium P (López-Ortega et al., 2013).

Not only crops may benefit from inoculation with P-solubilizing and NF microorganisms, but also organic fertilizers such as composts that could also be improved. Nutritional and microbiological qualities of composts can be improved by bacterial inoculation with NF bacteria (i.e., *A. chroococcum* and *A. lipoferum*; Kumar and Singh, 2001). Furthermore, compost N status improved due to the addition of a P-solubilizing bacterium (*Pseudomonas striata*) along with adequate P nutrition (Kumar and Singh, 2001). This is in accordance with field studies on fenugreek and soybean plants whose biomass, yield, and nutrient content increased in response to an integrated organo-mineral fertilization based on phospho-compost and farmyard manure inoculated with a microbial consortium that include *Rhizobium* sp., *Azotobacter* (as biological source of N) and numerous P solubilizing bacteria (Singh et al., 2010; Biswas and Anusuya, 2014). This is in agreement with a study by Shoghi-Kalkhoran et al. (2013) that evaluated the combined effect of biological fertilizers (farmyard manure), urea and inoculation with various PGPR (including *Azotobacter* and *Azospirillium* species) on grain yield, protein, fatty acids, and oil contents of sunflower crop. This integrated fertilization system improved sunflower productivity and seed oil quality and corroborates with other studies on root N absorption from chemical fertilizers that could be increased under application of both biological and manures fertilizers (Shata et al., 2007; Shoghi-Kalkhoran et al., 2013).

## Synergistic Use of Phosphorus and P-Solubilizing/Mobilizing Microorganisms

Combinatory use of PSM and P has been practiced and a number of studies evidenced improved agronomic efficiency of rock phosphate and P fertilizers such as, DAP, NPK, and TSP (Duarah et al., 2011; Kaur and Reddy, 2014; Adnan et al., 2017). For instance, Duarah et al. (2011) reported that application of both NPK fertilizer and a consortium of seven PSB strains selected for their high P solubilization properties (e.g., *Staphylococcus epidermidis*, *P. aeruginosa*, *Bacillus subtilis*, and *Erwinia tasmaniensis*) improved plants biomass and enhanced germination index in rice and cowpea bean owing to stimulation of specific enzyme biosynthesis such as amylase in seeds. Moreover, a recent study by Noor et al. (2017) evaluated the maize growth in response to impregnated DAP fertilizer with *Pseudomonas putida* (prepared by coating DAP (20 g/kg) with a mixture of organic material containing compost, molasses, and the *P. putida* bacterial strain). This study demonstrated the benefit of the combined DAP and PSB co-application as it improved maize dry matter (12%) yield and P uptake (33%) in addition to significant agronomic efficiency in terms of produced biomass that increased by 62% compared to unfertilized soil. Such a P fertilizer-bacteria alliance approach is peculiarly interesting in soil where P management is demanding.

**FIGURE 5:**
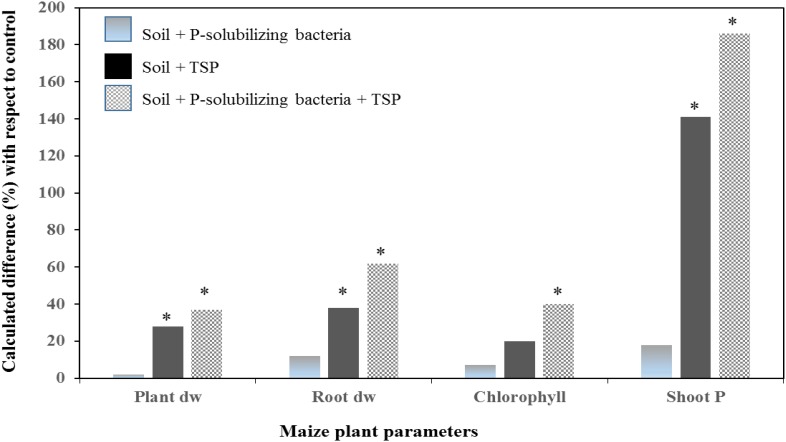
Effect of a Moroccan native P solubilizing bacteria on total plant dry weight, root dry weight, chlorophyll content and shoot P uptake, of maize (cv. SUPERMONARK) fertilized with TSP at 100 kg P_2_O_5_ ha^-1^ in calcareous soil (pH 8.2). Asterisks denote significant difference (*p* < 0.05) with regard to control.

**FIGURE 6:**
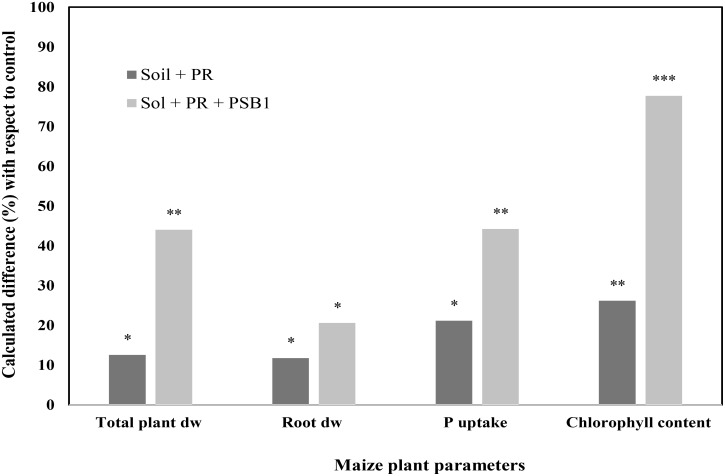
Effect of a Moroccan native P solubilizing bacteria (PSB1) on total plant dry weight, root dry weight, P uptake, and shoot chlorophyll content of maize (cv. SUPERMONARK) fertilized with rock phosphate (PR) at 250 kg ha^-1^ in calcareous soil (pH 8.2). Asterisks denote significant difference (*p* < 0.05) with regard to control.

Other findings illustrated in **Figures 5**, **6** reported an improvement of maize growth under combined treatment consisting of mineral P fertilizers (TSP – **Figure 5** and rock phosphate – **Figure 6**) and an efficient PSB strain. Those integrated treatments enhanced used of phosphate and TSP fertilization and improved maize plant agro-physiological performance including shoot P content, chlorophyll content, biomass of root and shoot. This is in agreement with findings by Kaur and Reddy (2015) who reported that fertilization with rock phosphate (59 kg P_2_O_5_/ha) and inoculation with two PSB strains induced high growth and yield performances (grains yield, shoot and root biomass, and P uptake) in wheat and maize. In addition, a recent study by Adnan et al. (2017) reported that P availability could significantly be improved under soil alkalinity conditions with the application of a multi-genera PSB inoculum (*Pseudomonas*, *Pantoea*, *Mycobacterium*, *Bacillus*, *Burkholderia*, *Arthrobacter*, and *Enterobacter*) supplied with mineral (single super phosphate and rock phosphate) and organic (poultry and farm yard manures) P fertilizers.

The positive impact of combining both AMF and P fertilizers on plant growth, development and P uptake has been studied. For instance, Cely et al. (2016) measured a significant agronomical response of cotton and soybean to a combined application of *Rhizophagus clarus* and NPK mineral fertilizer (200 and 100 kg/ha). The co-application “AMF/fertilizer” increased P uptake by 24% compared to the mineral fertilizer alone and that the mycorrhizal colonization remained unchanged in response to NPK application. This is in agreement with findings in **Figure 4** showing the impact of tow Moroccan native AMF strains (*Funneliformis mosseae* and *Rhizophagus intraradices*) on maize yield and symbiotic performance under mineral P (TSP, 130 kg/ha) fertilization. Increased P uptake and dry weight of shoots and roots was obtained in response to combined use of TSP and both AMF, and in particular with *F. mosseae* that exhibited higher arbuscular mycorrhizal colonization in the presence of TSP (**Figure 4**). Singh and Reddy (2011) also demonstrated the positive effect of *Penicillium oxalicum* and rock phosphate co-application on wheat and maize under alkaline conditions where plant P content and yield (70%) of maize were higher over the control. These results are consistent with findings by Yin et al. (2015) that application of two P-solubilizing fungal strains (*P. oxalicum* and *Aspergillus niger*) isolated from calcareous soil, enhanced P availability and effectively colonized maize roots which positively influenced aboveground biomass. Those responses were associated with higher biosynthesis of organic acids (i.e., acetic, citric, formic, lactic, malic, and succinic acids) when rock phosphate was added. More evidences through field and greenhouse experiments on the beneficial effects attributed to combinatory use of both PSM and P-based mineral fertilizers are highlighted in **Table 1**.

**Table 1:** Agronomical impacts of inoculation with P solubilizing microorganisms on various crops.

Crops	P solubilizing microorganisms	Experimental conditions and P fertilization	Agronomic impacts	Reference
Rice	*Gluconacetobacter* sp. *Burkholderia* sp.	Greenhouse Pot experiments. Fertilization: urea (90 kg N/ha), rock phosphate (60 kg P_2_O_5_/ha) or super phosphate (40 kg P_2_O_5_/ha) and murate of potash (11 kg K_2_O/ha).	Enhancement of PSB community higher phosphatase activity, increased P uptake and content, increased biomass, yield, number of panicles, and seeds/panicles.	Stephen et al., 2015
Rice	*Rahnella aquatilis Enterobacter* sp. *Pseudomonas fluorescens Pseudomonas putida*	Pot and field experiments. Fertilization : TSP (25, 75 and 150 kg/ha, K_2_SO_4_ (120 kg/ha, Urea 120 kg/ha).	Increased grain yield, biomass, number of stems/hill and panicles/hills.	Bakhshandeh et al., 2015
Maize	*Serratia marcescens Pseudomonas* sp.	Greenhouse and field experiments. Fertilization: urea 80 kg N/ha), SSP or rock P (20 kg P/ha).	Increased biomass (86%) and grain yield (64%) in field trials with higher P and N uptake.	Hameeda et al., 2008
Wheat	*Penicillium bilaji*	Pot and field experiments. Fertilization: rock P (20 kg P/ha) versus un-inoculated control (MAP 20 kg P/ha).	Increased plants biomass, P and Zn uptake.	Kucey, 1987
Maize Wheat	*Pantoea cypripedii Pseudomonas plecoglossicida*	Two years field experiments (seeds coated with bacteria). Fertilization: rock P (13 kg P/ha), urea (125 kg N/ha).	Increased maize grain (20%) and wheat grain (26%). Higher activity of dehydrogenase, alkaline and acid phosphatases and phytase enzymes.	Kaur and Reddy, 2014
Maize Wheat	*Pantoea cypripedii Pseudomonas plecoglossicida*	Two years field experiments. Fertilization: rock P (59 kg P_2_O_5_/ha), DAP (59 kg P_2_O_5_/ha).	Increased grain yield, P uptake, shoot and roots biomass compared to the DAP treatment.	Kaur and Reddy, 2015
Aloe vera	*Pseudomonas synxantha, Burkholderia gladioli, Enterobacter hormaechei Serratia marcescens*	Pot experiments in greenhouse. Fertilization: tricalcium phosphate. Inoculation with individual and mixture of all PSB.	Increased P availability and uptake. Increased Aloin-A content, leaves number and biomass.	Gupta et al., 2012
Potato	*Bacillus cereus Achromobacter xylosoxidans* And five other strains isolated from sweet potato roots.	Pot experiments in greenhouse (filled with soil/sand mixture 1:1 3 kg). Fertilization: rock P (4 g/pot).	Increased shoot and root biomass. Increased photosynthetic pigments.	Dawwam et al., 2013
Wheat Faba bean	*Aspergillus niger A. fumigatus Penicillium pinophilum*	Pot and column experiments. Fertilization: rock P or super TSP (15.5% P_2_O_5_).	Increased wheat yield by 32.8%. Increased faba bean yield by 29.4%. Increased P uptake.	Wahid and Mehana, 2000
Peanut	*Aspergillus niger Penicillium notatum*	Pot experiments Fertilization: tri-calcium P (20 mg P/kg).	Increased biomass and pod number.	Malviya et al., 2011
Common bean	*Aspergillus* sp. *Penicillium* sp.	Pot experiments Fertilization: rock phosphate (22.5 mg P/kg).	Increased biomass of shoot and root. Increased nodule number and biomass. Increased P uptake and N content.	Elias et al., 2016

All these positive and synergistic effects are in fact governed by complex mutli-factorial aspects, including soil nutrients status, overall pedoclimatic conditions, nature of the applied mineral P fertilizer, in addition to crop phenology and microorganisms/plant affinity. For instance in the specific case of AMF, numerous studies have shown P to be the most important element in mycorrhizal symbiosis regulation (Isobe et al., 2008; Schmidt et al., 2010; Smith et al., 2011). Moreover, recent use of next generation sequencing such as the 454-sequencing of the AMF *SSU* rRNA revealed AMF to be more diverse in P-based fertilization systems than previously described (Van Geel et al., 2015), which was solely based on morphological properties of the fungal spores. Other studies revealed that P is not the sole limiting factor in AMF development and that the remaining nutrients pathways are also as impactful. This was exemplified by Nouri et al. (2014) who reported promoting effect of N impairment prevails over the P effect, and suggested that AMF regulation depends on the complexes interactions of nutrients pathways. Moreover, crop phenology and soil depth, rather than P mineral fertilization, were found to be key factors in AMF effectiveness as demonstrated through a 3-year field study by Lui et al. (2016). This study highlighted AMF richness and distribution to occur at deeper soil layers and that optimal P application surprisingly increased AMF colonization of deeper maize roots.

## Multi-Trophic P- Solubilizing/Mobilizing and N_2_-Fixing Microorganisms Enhance P Use Efficiency

**FIGURE 7:**
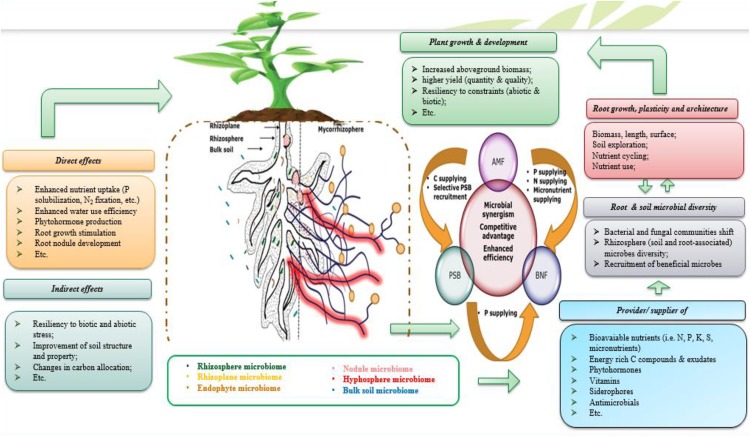
Simplified illustration of the microbial consortia concept highlighting beneficial rhizosphere PGPM and their direct (i.e., nutrients uptake via fixing N_2_, solubilizing P, producing plant growth promoting (PGP) substances like indole acetic acid, gibberellin, and cytokinin, etc.) and indirect effects (i.e., alleviation and/or protection against biotic and abiotic constraints) on root growth, symbiotic (rhizobial and mycorrhizal, etc.) and aboveground (plant growth, productivity, etc.) plant performance. Heterogeneity of the rhizosphere and root beneficial microbiome influences root growth and plasticity of root architecture that lead to effective exploration of soil and thus efficient nutrient uptake with positive consequences on growth and yield of the plant host.

It is a given that the objective of enhancing mineral fertilizer efficiency by exploiting microbial strategies, cannot be achieved unless we design, test and develop microbial formulations that prove its efficiency in a constant manner. One of the greatest challenges of the microbial inoculant field is to thrive in making use of efficient microbial consortium which is based on various microbial strain exhibiting various PGP properties and synergistically operating along with mineral resources as well as co-existence in interaction with plant hosts (**Figure 7**). Giving all knowledge provided above regarding the positive impacts that single inoculation may have on plant growth, productivity and nutrient use, it is also evidenced that adopting polymicrobial approach (**Figure 7**) could produce additional improvements as well as much more resiliency to contrasting conditions (Reddy and Saravanan, 2013; Meng et al., 2015; Hashem et al., 2016; Zhang et al., 2016). Following up on the importance of NF and P solubilizing/mobilizing microorganisms and the need to explore them together as a single polymicrobial component, this chapter deals with the positive interactions that may occur when combining use of the latter microorganisms concurrently with an efficient use of nutrients, notably P.

In this context, co-application of various PGPM including PSM, NF bacteria, AMF, and KSM, has been reported to yield better as compared to single inoculation (Rojas et al., 2001; Shen et al., 2016). Increased P and N uptake concurrently with improved yield of wheat plants were reported in response to positive interactive effects that occur when co-applying multiple rhizosphere microorganisms as a consortia-inoculant based on PSB (*P. striata*), NF bacteria (*A. chroococcum*), and AMF (*Glomus fasciculatum*) (Khan and Zaidi, 2007). Moreover, the specific interaction between PSB and NF bacteria is especially tangible in the case of legumes knowing that P availability is one of the most limiting factor during the N_2_ fixation process (**Figure 2**). In legumes, during formation and functioning of nodules, PSB may enhance P availability through the production of organic acids which provides evidence on synergism with the rhizobial symbiosis and so more when P mineral fertilizers are applied (Afzal et al., 2010). Similarly, when studying the associative effect of *Rhizobium* and PSB on chickpea yield, Alagawadi and Gaur (1988) reported an increased bioavailable P fraction, N uptake, and yield as a response to co-application of both strains under superphosphate fertilization, thus suggesting that co-applying nutrient-supplying microbes could halve chemical N input. On another hand, some plant species like *Piptadenia gonoacantha* cannot nodulate in the absence of mycorrhizal colonization and when P is a limiting factor and that AMF are likely to be involved in the BNF process as recently demonstrated by Júnior et al. (2017).

Besides dual inoculation with AMF and NF bacteria increased use of P, it was suggested that stimulation of other important physiological functions (such as nitrogenase activity, leghaemoglobin content, and micronutrients uptake such as Fe, etc.), are likely attributed to AMF involvement in mobilizing resources and alleviation of the adverse effects induced by alkaline stress (Abd-Alla et al., 2014). Noteworthy, aforementioned biotic association has proven advantageous in intercropping systems as demonstrated by Meng et al. (2015) that both AMF (*F. mosseae*) and *Rhizobium* (SH212) control N transfer in the soybean/maize intercropping system. Findings of the latter study provided evidences for a beneficial effect owing to a dual inoculation (AMF and *Rhizobium*) on enhanced nodule number, N uptake and transfer from soybean to maize, and improved maize biomass. Multiple positive responses were attributed to the role of AMF in N uptake, rhizosphere-induced changes (acidification by legume exudates) and enhanced competition toward deleterious microbes (Eckhard et al., 1995; Miransari, 2011; Song et al., 2015). Obviously, developing microbial consortia needs to take into consideration not only the functional aspect of the microbial strains and plant species, but also nutritional status and biochemical heterogeneity of soils. Nutrients limitations stimulate plant competition and complementary to niches and so for beneficial microbes whose physiological functions are to be impaired or promoted (Kuzyakov and Xu, 2013; Zhu Q. et al., 2016). For example, Zhang et al. (2016) concluded that AMF-PSB cooperation in terms of resources exchange (C and P) only occurred when additional P was supplied leading to enhanced hyphae elongation and shoot P content. On the opposite, under limiting P conditions, AMF development was severely hindered which was attributed to competition factors.

In addition to its contribution in nutrient availability, uptake and yield improvement, microbial cooperation could truly shine under adverse biotic and abiotic conditions, and in particular where nutrient use efficiency is impaired due to altered plants physiological and metabolic process. For example, beneficial traits provided by the AMF/Rhizobia association which is the most studied tripartite symbiotic association were reported in several studies (i.e., Biró et al., 2000; Mirdhe and Lakshman, 2009; Awasthi et al., 2011; Reddy et al., 2016; Zhu R.F. et al., 2016). Inoculation of faba bean with microbial consortia comprising both *R. leguminosarum* and a mixture of AMF strains (*Acaulospora laevis*, *Glomus geosporum*, *Glomus mosseae*, and *Scutellospora armeniaca*) under four level of alkalinity (pH: 7.5, 8, 8.5, and 9) resulted in improved nodulation (higher number and biomass) compared to single-inoculated plants that were substantially inhibited by the alkaline conditions. The concept of microbial consortia could also prove beneficial with regards to other constraints such as salinity, one of the major issue faced by today’s agriculture knowing that virtually 20% of irrigated croplands are concerned (Negrao et al., 2017). In this context, fertilizers efficacy cannot be optimal since all the plant vital process (i.e., photosynthesis, energy, protein synthesis, and lipid metabolism, etc.) are heavily debilitated (Parida et al., 2005). In this regards, Hashem et al. (2016) demonstrated that interaction between AMF (*F. mosseae, R. intraradices*, and *C. etunicatum*) and a salt-tolerant (*B. subtilis*) significantly promoted plant growth when subjected to salinity constraint. This salt-tolerant tripartite symbiosis enhanced nodulation, dry biomass, P content, leghemoglobine, and protein content of *A. gerrardii* compared to untreated and mono-inoculated plants. Hashem et al. (2016) explained resilience to salinity to be attributed to *B. subtilis* which indirectly promoted nutrient acquisition via a better AMF colonization. Similarly, inoculation of two salt-treated maize varieties with two PGPR (*Rhizobium* sp. and *Pseudomonas* sp.) led to a higher proline production and a lower osmotic potential concurrently with enhancing P uptake and dulling detrimental effects induced by salinity (Bano and Fatima, 2009). Also, positive effects on osmotic adjustment under salinity and drought constraints have been associated with ectomycorrhizal colonization (Li et al., 2012). Under low P availability, naturally formed mycorrhizal symbioses or through genetically modified crops may thrive by inducing organic acids exudation which was shown to be a particularly valuable trait if accompanied by the ability to release compounds that prevent any further microbial breakdown of organic acids in the rhizosphere (Tomasi et al., 2008; Smith and Smith, 2011). Another example is that enhanced P availability may also improve tolerance to toxic elements, as indicated by Yao et al. (2014) findings that cadmium-treated roots of *Trifolium* sp. produced polyphosphate which chelated cadmium in the mycorrhizal hyphae of *Rhizophagus irregularis* concurrently with improved fitness for both plant and fungal partners. Overall, highly valuable mycorrhizae biotechnological applications are well-known, especially for tree improvement. However, significant progress is still lacking regarding the use of such a multifunctional mycorrhizae fungi for improving yield of major crops such as protein-rich grain legumes that hold valuable promises for the agriculture of tomorrow.

## Future Perspective and Challenges of Microbial-Based Agro-Inputs

As global warming is becoming a reality endangering nutritional demand, there is a need for innovative agro-inputs that enable agriculture to adapt to worsening environmental situations and exploiting microbial resources is one of the most promising solution to achieve such aim. Indeed, it is clear today that microbial inoculants, a sub category of the so-called biostimulants, have become one of the attractive agro-inputs for sustainable intensification of agriculture, especially for smallholders (Du Jardin, 2015). Biostimulants have gained substantial ground market wise, owing to the impressive know-how acquired during the last two decades, and most importantly to the involvement of low-cost technologies in their production process. However, despites all the aforementioned conveniences and numerous scientific and field evidences of their agronomic effectiveness, efforts are still required to make them full-fledged commodities that are used as standard by farmers.

There is a growing body of evidences about the large number of microbes that have been found to be highly beneficial for soil fertility and plant productivity in many major cropping systems. At the same time, many reports have demonstrated inconsistent and poorly repeatable results via controlled and field trials (Bhattacharjee et al., 2008; Martinez-Viveros et al., 2010), which may indicate uncertainty in the efficacy of the microbial inoculants that should be aligned with intricate biotic and abiotic factors including plant species, native microbial communities, environmental conditions, soil type and soil-related management practices such as fertilization, cropping systems, irrigation, and biocontrol strategies (Pereg and McMillan, 2015). Progress in this area would ultimately depend on a clear understanding of the latter factors in order to guarantee a successful manipulation of agriculture microbes, their commercialization, and widespread use. This is in agreement with the saying “big potential in small packages” by Matt Kleinhenz (Third world congress on the use of biostimulants in agriculture 2017, Miami) who portrayed the current state of the microbial-based biostimulants whose development presumably rely on coping with several issues relatively to both technical and economic aspects. Another concern is arguably related to misconceptions and lacking objectives in terms of research programs development as most research works are driven by “substitution approaches” where microbial inoculants are labeled as direct competitors to well-established agro-inputs with proven efficacy such as fertilizers.

Next generation agriculture should henceforth make use of all available resources and designing novel agro-models that focus on how to achieve perfect alliance between biologicals, chemicals, and biocomputing technologies. In that regard, adopting multidisciplinary approaches in developing microbial-based solutions concurrently with mineral fertilizer resources is paramount as it could lead to creating market’s opportunities and new agricultural paradigms based on new concept of sustainability, which is in tune with contemporary’s conceptions of today’s individuals. In this regards, scientists and manufactures interested in microbial-based biostimulants should focus on delivering stable formulations capable of withstanding harsh storage conditions and guaranteeing extended shelf life of active ingredients through limiting viability loss. Most importantly, microbial formulations must be compatible with conventional agro-equipment and other agro-inputs, especially mineral fertilizers, so their supply chains could be aligned. As a matter of fact, formulation is one of the most critical step in microbial inoculants manufacturing and several carriers have been used with contrasted results depending on the microbial species and pretreatment methods. Those carriers mostly include organic materials (i.e., peat, lignite, and composts, etc.) and polymeric compounds (i.e., alginate, agar, pectin, and chitosan, etc.; Bashan et al., 2014). Multi- and inter-disciplinary approaches are worth considering when designing innovative microbial formulations. This will open up new insights into an unexploited research area such as combining new-generation coating and microbial technologies that likely should arouse particular interests to innovative smart fertilizers. For instance, microbial biotechnologies would benefit from other emerging technologies such as those related to EEF and controlled release fertilizers, tough not largely used for staple crops and costly to be applied for an intensive agriculture (Shaviv, 2000; Trenkel, 2010; Linquist et al., 2013). For example, recent advances in coating technology that have led to the development of new-generation fertilizers particularly aiming at improving N use efficiency (reducing leaching, volatilization, and denitrification) may be exploited to enhance P fertilizers efficiency and uptake. This would contribute overcoming common issues related to low P availability which is pH-dependent, readily bounded with divalent cations and belowground leached, thus precise release rate and efficient plant root P uptake may be achieved. That being said, to our knowledge little has been done regarding production of customized carriers able to respond to all required quality criteria. For instance combining new-generation coating and microbial technologies is an unexploited research area that should arouse more interests. Breakthrough in that department could be a true game changer, thus giving rise to innovative smart fertilizers, matching the few concepts that precision agriculture relies on (sensing technology, farming satellite, data analysis, and controlled release fertilizers, etc.) while providing possibilities to enhance specific microbial biological functions related to nutrient dynamic in soils.

Given altogether, developing strategies relying on understanding potential modes of actions that provide possibilities to enhance specific microbial functions related to nutrient dynamic in soils, strengthening scientific and industrial collaborative partnerships, meeting farmers’ requirements are considered paramount in conceiving targeted products and answering specific consumer needs. Fostering proximity to growers should be given a special consideration since farmers’ acceptance has to be the utmost priority that can only be achieved through in-field demonstrations, producing reports and data specifically tailored for growers’ specificities. In addition, needless to say that the triumph of the next generation of agro-inputs based on microbial inoculants is largely dependent on regulatory clearness and adopting collaborative mindset where progress is made through farmers, scientists from private and public research institutes, advisers and policy makers. This will help moving toward integrated and profitable ecosystems where all inputs are managed following wholesome principles and aiming at optimizing nutrient use efficiency in a context where climate variability is persistently threatening for food productivity.

## Concluding Remarks

There are two main reasons for the need of efficacious microbial strategies concurrently with efficient mineral P fertilization. The first reason, providing they are unavoidable to crop productivity, is that use of mineral fertilizers with higher eco-efficiency would definitely increase as to meet future needs of the growing world population. The second is to ensure sustainable agricultural productivity while providing valuable ecosystem services through optimized microbial rhizosphere activities such as BNF, P solubilization, and overall nutrients dynamic by staple crops including legumes and cereals. Currently, multidisciplinary approaches have been adopted worldwide taking advantage from all available data and overwhelming progress in plant and microbial biotechnologies, and in particular to make use of nutrient resources efficient and sustainable. As per current knowledge on the evident roles of beneficial microbes (i.e., NF, P-solubilizing, and -mobilizing, etc.) in plant nutrition, soil fertility and stress tolerance, new routes and perspectives based on multi-disciplinary approaches should be considered in order to advance testing efficacious microbiological resources within profitable integrated plant nutrient agro-systems.

Indeed, continuous designing, developing and testing microbial-based formulations to be used as a component in efficient IPNMS has gained worldwide interest in recent decades and so more under the ever-increasing global demand for food production. Diverse microbial groups, with focus on NF and P-supplying microbes detailed in this review paper, are agriculturally beneficial and their contribution in agriculture does not aim only to improve specific biological functions (either directly or indirectly), but most importantly to increase availability and plant uptake efficiency of major soil pool nutrients and ultimately increase the eco-efficiency in use of mineral fertilizers. One of the most promising mean is to strengthening research on innovative IPNMS which include a variety of multi-functionally biotic and abiotic resources, particularly highly efficient PGPM in combination with mineral fertilizers. The right combination of resources (mineral and microbial), right rate of resources, right application time, right plant host, adequacy to soil and climatic conditions, and positive legacy effects are highly essential for decisions on relevant nutrient formula, cropping systems as well as best management practices that will lead to enhance crop productivity and soil fertility.

Furthermore, in order to achieve those goals with particular emphasis on P fertilizers as described in the paper, a good understanding of the tripartite interaction between microbial inoculants, mineral fertilizers, and crop species is a prerequisite. In fact, research is making recently great progress in that department through advances in several plant–microbe-related research areas including genomics, metabolomics and phenomics. In this context, high throughput technologies would provide necessary data enabling a better understanding of the intricate interactions within the holobiome, more particularly to unravel behavior responses of beneficial microbes in agroecosystems. Taking altogether, it is evident that current advances in terms of plant-, microbe-, and soil-focused research have led to developing crops with specific traits and fine-tuned for higher nutrient uptake and tolerance to multiple constraints. Likewise, these traits have also been targeted in microbes and biotechnological progresses thrived to provide beneficial microbes with proven efficiency. Now, securing sustainable higher yield and productivity in the near future will rely on exploiting all available multidisciplinary progresses in order to design innovative crop–microorganisms biosystems with synergistic and complementary interactions.

## Author Contributions

All authors equally contributed to the preparation of the review, revised the text at different stages of the writing process, and read and approved the current manuscript.

## Conflict of Interest Statement

The authors declare that the research was conducted in the absence of any commercial or financial relationships that could be construed as a potential conflict of interest.
